# Finite element analysis comparison between superior clavicle locking plate with and without screw holes above fracture zone in midshaft clavicular fracture

**DOI:** 10.1186/s12891-019-2847-y

**Published:** 2019-10-22

**Authors:** Nachapan Pengrung, Natthaphop Lakdee, Chedtha Puncreobutr, Boonrat Lohwongwatana, Paphon Sa-ngasoongsong

**Affiliations:** 1 Department of Orthopedics, Faculty of Medicine Ramathibodi Hospital, Mahidol University, 270, Rama VI Road, Ratchathewi, Bangkok, 10400 Thailand; 2 Biomechanic Research Center, Meticuly Co. Ltd., Chulalongkorn University, Bangkok, Thailand; 3 Biomedical Engineering Research Center, Chulalongkorn University, Bangkok, Thailand; 4 Department of Metallurgy Engineering, Faculty of Engineering, Chulalongkorn University, Bangkok, Thailand

**Keywords:** Superior clavicle locking plate, Midshaft clavicular fracture, Plate fixation, Screw hole, Implant failure, Peak stress

## Abstract

**Background:**

Midshaft clavicular fractures are common fractures and generally treated conservatively. Among the surgical options, plate fixation is the most popular and has been biomechanically and clinically proven in numerous studies. However, implant failures caused by plate deformations or breakage still occur in up to 16.7% of cases, and recent studies showed that screw holes above fracture zone (SHFZ) might be the at-risk location. Using finite element analysis, this study aimed to test the biomechanical property of the superior clavicle locking plate (SCLP) with and without SHFZ in comminuted midshaft clavicular fracture.

**Methods:**

Finite element models of comminuted midshaft clavicular fracture fixed with standard 8-hole titanium SCLP with screw holes (SHFZ plate) and without screw holes above fracture zone (No-SHFZ plate) were built. Both groups were tested under three different loading models (100-N cantilever bending, 100-N axial compression, and 1-Nm torsion). The average peak stress on medial clavicle, fracture zone, and lateral clavicle, and the peak stress on each screw hole (or the same position in the No-SHFZ plate) were measured and compared.

**Results:**

The highest average peak stress on the fracture zone was higher than those on medial and lateral clavicles under all loading conditions in both plates. However, the No-SHFZ plate significantly reduced the average peak stress value on the fracture zone, compared to the SHFZ plate (45.0% reduction in cantilever bending, 52.2% reduction in axial compression, and 54.9% reduction in axial torsion). The peak stress value on the maximal stress point in the SHFZ and No-SHFZ plates with cantilever bending, axial compression, and torsion loads were 1257.10 MPa vs. 647.21 MPa, 186.42 MPa vs. 131.63 MPa, and 111.86 MPa vs. 82.41 MPa, respectively.

**Conclusion:**

The weakest link of the SCLP construct in comminuted midshaft clavicular fracture fixation is the SHFZ, especially in the cantilever bending load. Additionally, the biomechanical property of the SCLP without SHFZ model (No-SHFZ plate) is superior to the standard SCLP model (SHFZ plate), with a significantly lower peak stress on the SHFZ location in all loading conditions. We recommend a new SCLP design with SHFZ to prevent implant failure and improve surgical outcomes.

## Background

Clavicle fractures are one of the most common around the shoulder girdle, accounting for 2.6% of all fractures and 44% of shoulder girdle fractures. Notably, the majority of clavicle fractures occur at the midshaft of the clavicle (81%), which associates with displacement in 48% of cases and with comminuted patterns in 19% [1]. These fractures are generally treated conservatively [2], but those with severely displaced or comminuted patterns have high risk for delayed union or nonunion and are indicated for surgical treatment [3–5]. Among the surgical options for treating midshaft clavicular fractures, plate fixation—either superior or anteroinferior placement—continues to be the most popular surgery due to the plate’s excellent biomechanical strength [6] and reliable clinical outcomes [5]. However, postoperative complications with implant failures, caused by plate deformation or breakage, have still required revision surgery in as many as 6.9–16.7% of plate fixations [7–10], especially when wedge or comminuted fractures have been present due to the direct negative impact on the fixation stability [11]. A previous study identified two risk factors for plate breakage: the use of a reconstruction plate and the bridging plate technique [7]. Moreover, a recent finite element analysis study on clavicle fracture fixation showed that the maximum stress in the precontoured superior reconstruction plate fixation without lag screws occurred at the edge of screw holes above fracture zone (SHFZ) [12]. To the best of our knowledge, no previous study has evaluated the biomechanical response of the superior clavicle locking plate (SCLP) without SHFZ. We hypothesized that a new SCLP design without SHFZ would have better biomechanical behaviors for comminuted midshaft clavicular fracture fixation than the standard SCLP (with SHFZ) in terms of the stress distribution under mechanical load. Therefore, the aim of this study was to perform a comparative stress analysis between the SCLP with and without SHFZ in comminuted midshaft clavicular fracture fixation.

## Methods

This study has been reviewed and approved by the Institutional Review Board at Mahidol University, based on the Declaration of Helsinki (COA no. MURA2018/964, Protocol number 11–61-74).

### Finite element model

**Fig. 1:**
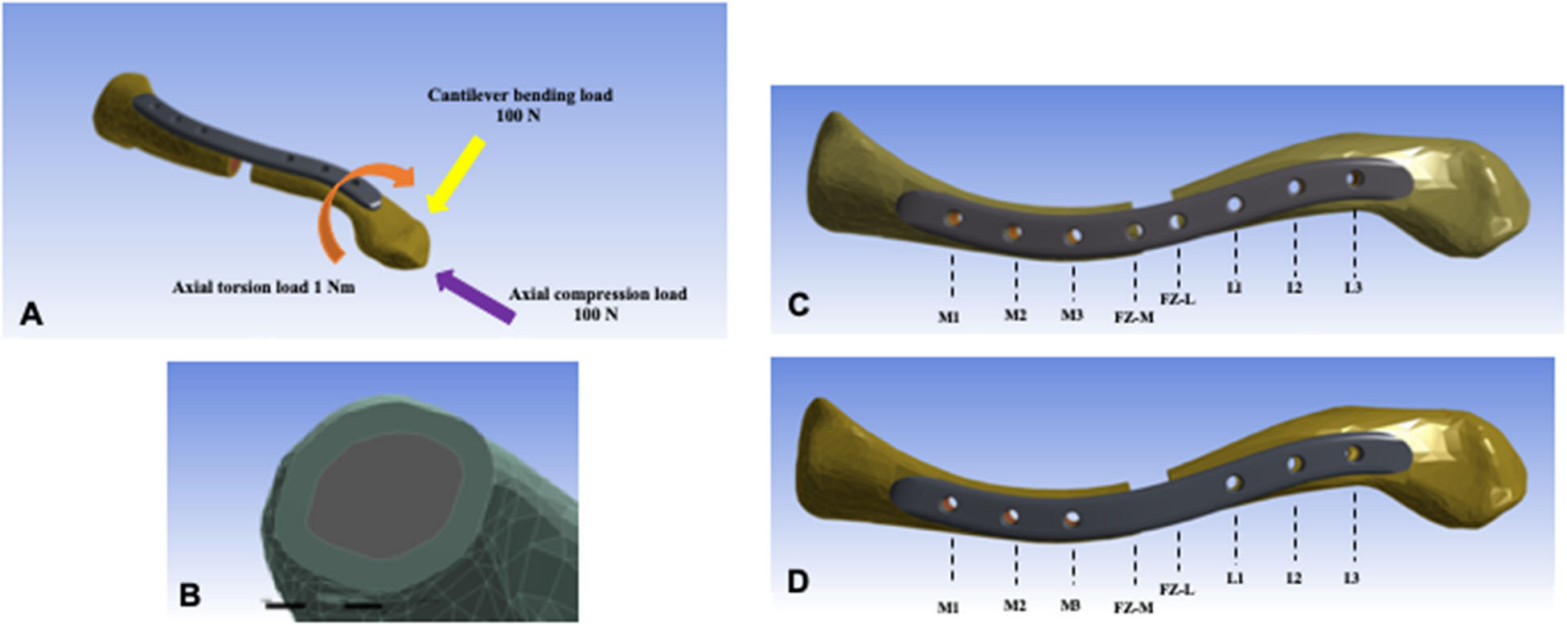
Finite element model, the loading with the boundary conditions applied in current study (**a**), the cross-section view of the finite element model (**b**), and the reference points on the SHFZ plate (**c**) and the No-SHFZ plate (**d**)

The 3D-CAD model in this study was from the commercially available high-resolution DICOM file of large-sized left clavicle (Sawbones Vashon, WA, USA); the model was imported into Ansys 19.2 software (Ansys, Inc., Canonsburg, PA, USA) for the finite element (FE) analysis. The FE meshes were generated as a tetrahedral 2.0 mm size for bones, a tetrahedral 1.5 mm size for plates, and a hexahedral 2.0 mm size for screws. The average mesh quality was 0.81. The SCLP model was a left-sided, 8-hole titanium LCP superior clavicle plate 3.5 mm (Synthes, Solothurn, Switzerland), but used only locking holes instead of the pre-existing Combi holes (Fig. 1a). A 2-mm uniform thickness was set for the outer layer for the cortical bone with the remaining inner layer for cancellous bone (Fig. 1b). The fracture site was simulated by creating a 10-mm gap at the middle of the clavicle to represent a comminuted midshaft fracture. This fracture model was then fixed with six 3.5-mm locking screws on both medial and lateral clavicle fragments without screw fixation in two holes above the fracture zone (SHFZ plate, Fig. 1c). In another model, these holes were erased and filled with titanium alloy materials matching the plate materials (No-SHFZ plate, Fig. 1d). The contact interface between all items was set as a 0.4 frictional coefficient for the bone-plate interface and set as totally bonded for the plate-screw and screw-bone interfaces. The material properties were set up using literature references [13] (Table 1).

**Table 1:** Material properties utilized in the finite element model

Materials	Young’s modulus (MPa)	Poisson’s ratio
Cortical bone	17,000	0.3
Cancellous bone	1000	0.3
Titanium alloy	9.60E+ 10	0.36

### Mechanical loading simulation and outcome measurement

As a result of fully constraining the medial end of the clavicle, three common loading modes with boundary condition, mirroring those in a previous study by Huang et al. [14] —100 N of cantilever bending, 100 N of axial compression, and 1 Nm of clockwise axial torsion—were respectively applied at the lateral end of the clavicle (Fig. 1a).

Outcomes were measured as the equivalent stress or von Mises stress in megapascal (MPa) on eight reference points at the eight screw hole locations in the SHFZ plate or the same position in the No-SHFZ plate (M1, M2, M3, FZ-M, FZ-L, L1, L2, and L3) (Fig. 1c-d). The peak stress on each reference point was defined as the highest von Mises stress at each location. The average peak stress was defined as the mean peak stress from three different areas: 1) medial clavicle (M1, M2, and M3), 2) fracture zone (FZ-M and FZ-L), and 3) lateral clavicle (L1, L2, and L3).

## Results

**Fig. 2:**
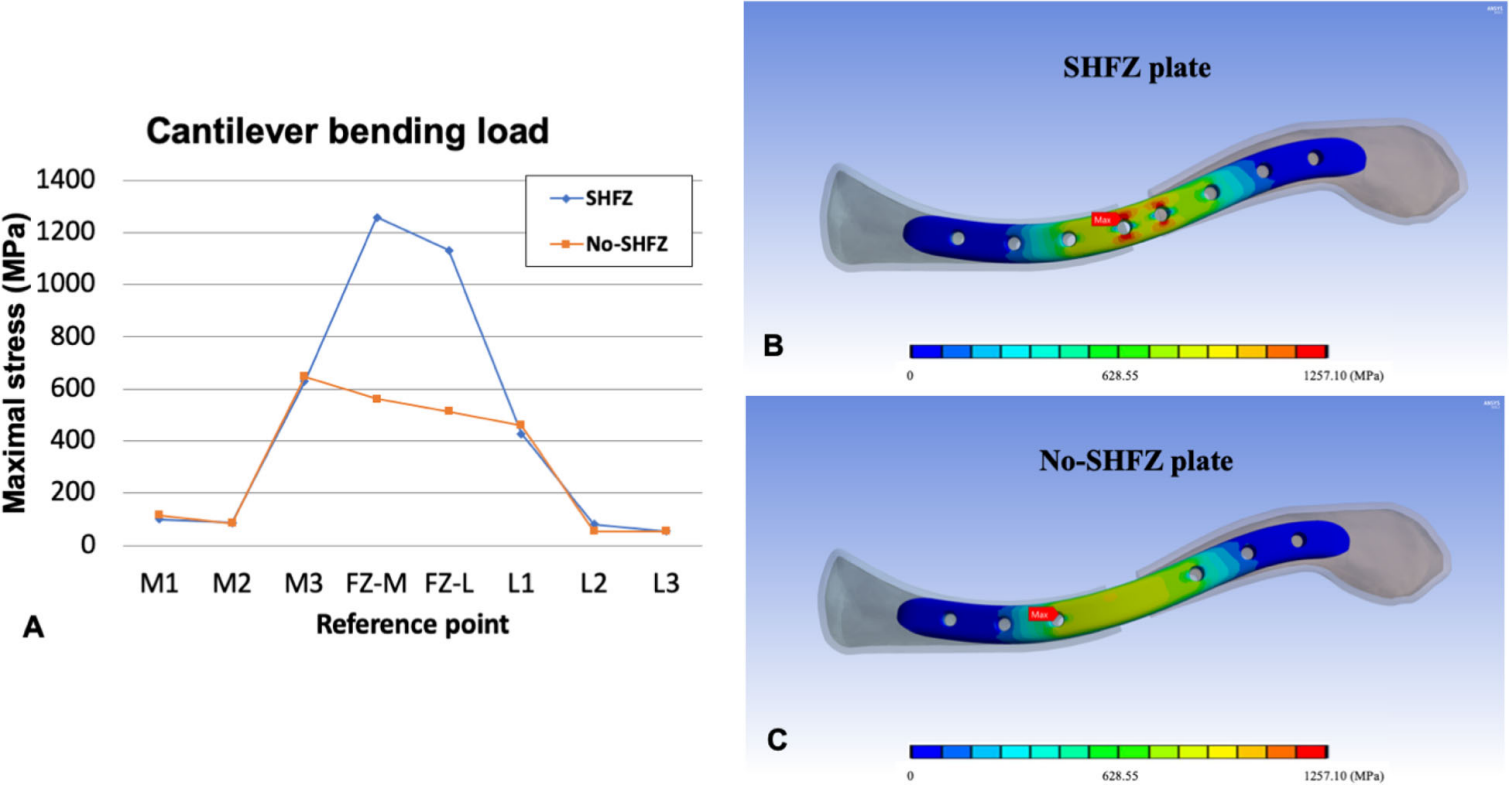
Illustration of peak stress value on each reference point (**a**) and von Mises stress pattern comparison between the SHFZ plate (**b**) and the No-SHFZ plate (**c**) from cantilever bending load simulation

**Fig. 3:**
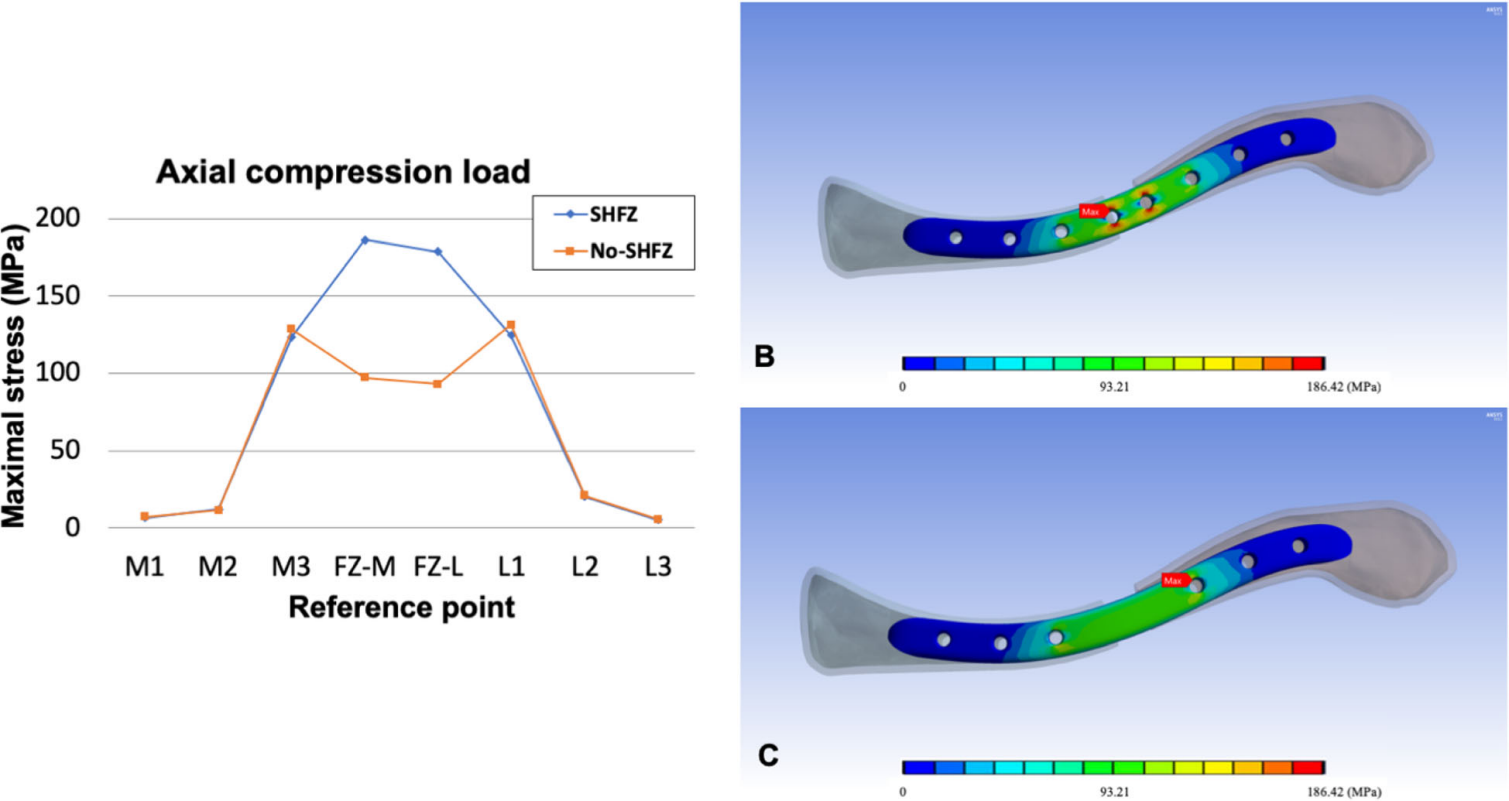
Illustration of peak stress value on each reference point (**a**) and von Mises stress pattern comparison between the SHFZ plate (**b**) and the No-SHFZ plate (**c**) from axial compression load simulation

**Fig. 4:**
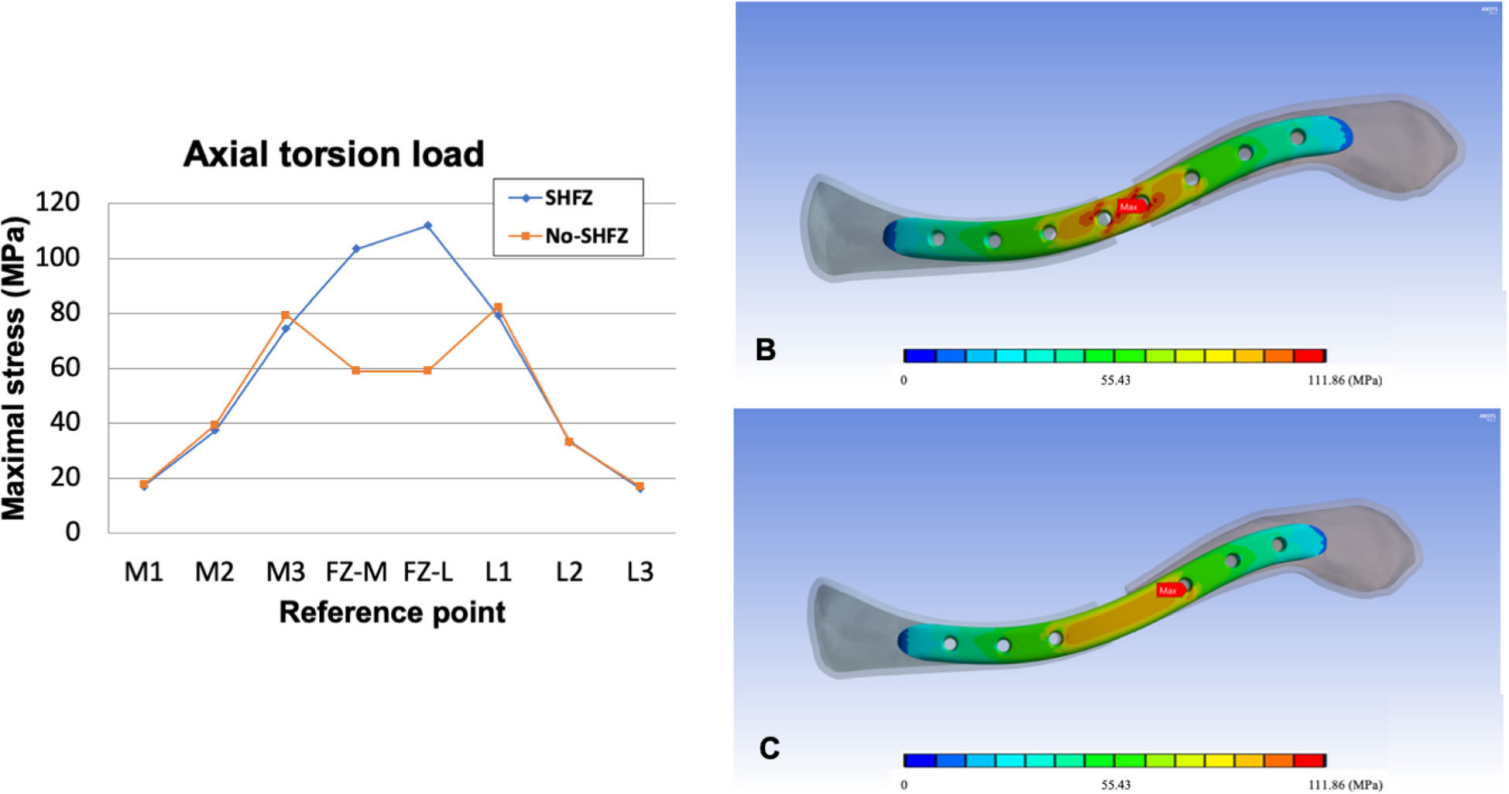
Illustration of peak stress value on each reference points (**a**) and von Mises stress pattern comparison between the SHFZ plate (**b**) and the No-SHFZ plate (**c**) from axial torsion load simulation

The details of von Mises stress values in both plates at each reference point in all three loading conditions are shown in Table 2. The comparison of peak stress values (Figs. 2a, 3a, and 4a) and the von Mises stress patterns between the SHFZ and No-SHFZ plates in cantilever bending (Fig. 2b-c), axial compression (Fig. 3b-c), and axial torsion (Fig. 4b-c) loads are illustrated in Figs. 2, 3, and 4, respectively.

**Table 2:** Comparison of the peak stress values and the normalized von Mises stress pattern between the SHFZ and No-SHFZ plates in three different loading conditions

Reference point^a^	Peak von Mises stress value (MPa)					
	Cantilever bending load		Axial compression load		Axial torsion load	
	SHFZ plate	No-SHFZ plate	SHFZ plate	No-SHFZ plate	SHFZ plate	No-SHFZ plate
M1	100.48	116.63	6.60	7.40	17.13	17.54
M2	88.18	84.84	12.35	11.54	37.39	39.44
M3	628.77	647.21 ^Max^	123.89	128.61	74.52	79.47
FZ-M	1257.10 ^Max^	561.30	186.42 ^Max^	97.30	103.42	59.07
FZ-L	1131.80	514.42	178.72	93.19	111.86 ^Max^	59.09
L1	428.44	461.05	124.78	131.63 ^Max^	79.19	82.41 ^Max^
L2	81.74	54.76	20.47	21.03	33.23	33.09
L3	55.34	54.76	5.21	5.75	16.20	16.86
Average peak stress^b^						
Medial clavicle (M1–3)	358.48	282.89	47.61	49.18	43.01	45.48
FZ (FZ-M, FZ-L)	1194.45	537.86	182.57	95.25	107.64	59.08
Lateral clavicle (L1–3)	188.51	190.19	50.15	52.80	42.87	44.12

^a^Reference point: the point of each screw hole (SH) position in the SHFZ plate, or the same position in the No-SHFZ plate, from medial end to lateral end

M1, M2, and M3: the 1st, 2nd, and 3rd medial SH positions of the medial clavicle fragment

FZ-M and FZ-L: the medial and lateral SH positions, or the same ones in the No-SHFZ plate, above the fracture zone

L1, L2, and L3: the 1st, 2nd, and 3rd medial SH positions on the lateral clavicle fragment

^b^Average peak stress: the mean of peak stress value in each zone (medial clavicles include M1, M2, and M3; SHFZ includes FZ-M and FZ-L; and lateral clavicle include L1, L2, and L3)

^Max^: the presented reference point has the highest peak von Mises stress value in that loading condition

### Stress distribution and maximal stress point in cantilever bending

Under 100 N of cantilever bending load, the average peak stress on the medial clavicle (M1, M2, and M3), fracture zone (FZ-M and FZ-L), and lateral clavicle (L1, L2, and L3) in the SHFZ plate was 272.48 MPa, 1194.45 MPa, and 188.51 MPa, respectively. Conversely, the avereages in the same area of the No-SHFZ plate were 282.89 MPa, 537.86 MPa, and 190.19 MPa, respectively. The maximal stress point from this bending load in the SHFZ plate was located at the FZ-M location (1257.10 MPa); however, the maximal stress point in the No-SHFZ plate was at the M3 location (647.21 MPa) (Table 2 and Fig. 2a-c).

### Stress distribution and maximal stress point in axial compression

The average peak stress from 100 N of axial compression load on the medial clavicle, fracture zone, and lateral clavicle in the SHFZ plate was 47.61 MPa, 182.57 MPa, and 50.15 MPa, respectively. Meanwhile, the averages in the same area of the No-SHFZ plate were 49.18 MPa, 95.25 MPa, and 52.80 MPa, respectively. The locations of the maximal stress points in the SHFZ and No-SHFZ plates were at the FZ-M location (186.42 MPa) and L1 location (131.63 MPa), respectively (Table 2 and Fig. 3a-c).

### Stress distribution and maximal stress point in axial torsion

With 1 Nm clockwise axial torsion load, the average peak stress on the medial clavicle, fracture zone, and lateral clavicle in the SHFZ plate was 43.01 MPa, 107.64 MPa, and 42.87 MPa, respectively. Average peak stress in the No-SHFZ plate was 45.48 MPa, 59.08 MPa, and 44.12 MPa, respectively. The maximal stress point from this axial torsion load in the SHFZ plate was located at the FZ-L location (111.86 MPa); however, in the No-SHFZ plate, the maximal stress point was at the L1 location (82.41 MPa) (Table 2 and Fig. 4a-c).

## Discussion

In the present study, the finite element analysis was developed to compare the biomechanical behavior between SLCP with SHFZ (SHFZ plate) and without the SHFZ (the No-SHFZ plate) to better understand the effect of SHFZ in the SLCP construct on the treatment of comminuted midshaft clavicular fractures).

Regarding the SHFZ plate, our main findings were that the average peak stress on the fracture zone (FZ-M and FZ-L) was higher than the average stress on the medial (M1–3) and lateral clavicle (L1–3) in all three loading conditions. Moreover, we found that the peak von Mises stress on the reference points nearest to the SHFZ (M3 and L1) were much higher than the other reference points for the medial and lateral clavicle (M1–2 and L2–3) in all three loading conditions. Concerning the effect of different loads on the fracture zone, the average peak stress from the cantilever bending load (1194.45 MPa) was also much greater than the peak stress from the axial compression and axial torsion loads (182.57 MPa and 107.64, respectively). The highest peak stress value (maximal stress point) from the cantilever bending condition occurred at the FZ-M location in the SHFZ plate, as 1257.10 MPa (Table 2). These results imply that the screw holes above the comminuted fracture zone (FZ-M and FZ-L) have the greatest risk for implant failure in all loading conditions, especially in cantilever bending. The results also support the findings from previous research that using superior plate fixation without screw insertion on the fracture zone in the treatment of midshaft clavicle fracture, as a bridging plate with screw holes above the fracture zone similar to the SHFZ plate in our study—was one risk factor for construct failure [7]. Likewise, our findings also support the results from previous studies that the insertion of the screw head into the empty locking screw holes above the fracture zone could augment the plate’s biomechanical property as it improves the bending stiffness and extends the fatigue life of the locking plate [15], with significantly higher fatigue strength [16]. However, to our knowledge, this effect of the augmentation technique might not apply in every case, especially in titanium plate fixation or when using combination locking-compression holes [16, 17].

Comparing the SHFZ and No-SHFZ plates, the results of this study showed that the No-SHFZ plate more significantly reduced the average peak stress value on the fracture zone (45.0% reduction in cantilever bending, 52.2% reduction in axial compression, and 54.9% reduction in axial torsion). We also found that the maximal stress points from the loading conditions in the No-SHFZ plate were not located at the SHFZ reference points (FZ-M and FZ-L locations) but instead located at the reference points nearest to the SHFZ (M3 in cantilever bending and L1 in both axial compression and axial torsion) (Table 2). However, these values were nearly the same as the corresponding reference points in the SHFZ plate (M1–3 and L1–3) (Table 2 and Figs. 2, 3 and 4), which could imply that the No-SHFZ plate does not significantly affect the stress—with respect to the stress magnitude and the stress distribution—from different loads in these locations. Therefore, we suggest that, in the treatment of comminuted midshaft fracture, a new plate design without SHFZ that increases the plate thickness in the area of all at-risk reference points could potentially prevent implant failure.

The limitations of this study included the assumption of the ideal bonded construct that uses only one standard clavicle model fixing with the same SCLP implant geometry and applying only the constant unidirectional force for all loading conditions. Although these simplifications were helpful for comparing the SHFZ and No-SHFZ plates, we did not analyze some errors—such as micromotion at bone-plate interface and the stress riser effect of the screws, variation of clavicle morphology and bone quality, and real-life loading force on the clavicle from the combined muscular and external forces [12]. However, regarding the avoidance of these errors, we believe our results would be applicable for most comminuted midshaft clavicular fracture patients with good bone quality. Moreover, the role of using 3D-printing technology for patient-specific anatomical implants in orthopaedic trauma has been increasing recently [18], which could allow for manufacturing anatomical No-SHFZ plates for patients, especially for small-sized clavicles, such as in Asian patients [19]. Nevertheless, further studies, such as biomechanical testing and clinical studies, are needed to verify the results of this study.

## Conclusion

Our findings showed that the screw holes above fracture zone (SHFZ) are the weakest link of the superior clavicle locking plate (SCLP) construct in the comminuted midshaft clavicular fracture fixation, especially in the cantilever bending load. Moreover, the SCLP without the SHFZ model (No-SHFZ plate) demonstrated better biomechanical behaviors than the standard SCLP model (SHFZ plate) by lowering the stress on the SHFZ location under all loading conditions. Therefore, based on our findings, a new SCLP design without SHFZ is recommended to prevent implant failure when treating midshaft clavicular fractures via plate fixation and to improve surgical outcomes. However, mechanical tests and clinical trials are required to validate and enhance this concept.

## Data Availability

Supporting data is available with the corresponding author on request.

## Abbreviations

SCLP: superior clavicle locking plate
SHFZ: screw holes above fracture zone
